# Combined Effects of Potato Peel Powder and Pomegranate Peel Powder on the Quality Attributes of Refrigerated Chicken Meatballs

**DOI:** 10.3390/foods15173069

**Published:** 2026-08-29

**Authors:** Basma R. Abdel-Moatamed, Mohamed H. H. Roby, Mohamed Mahmoud Shaban

**Affiliations:** Department of Food Science and Technology, Faculty of Agriculture, Fayoum University, Fayoum 63514, Egypt

**Keywords:** potato peels, pomegranate peel, natural extracts, chicken meatballs, quality attributes, antimicrobial, antioxidant activity

## Abstract

This study investigated the effectiveness of potato peels and pomegranate peel powder as natural preservatives to improve the quality of chicken meatballs. A total of seven treatment formulations were evaluated, comprising one control and six peel-powder-supplemented formulations. Chicken meatball formulations were prepared by incorporating potato peels and pomegranate peel powder at different levels: a control (without peel powder), 2% potato peels, 2% pomegranate peel, 4% potato peels, 4% pomegranate peel, a combination of 2% potato peels + 2% pomegranate peel and 4% potato peels + 4% pomegranate peel. The physicochemical, microbiological, and sensory characteristics of the products were evaluated during 20 days of refrigerated storage at 4 ± 1 °C. Furthermore, bioactive compounds were extracted from potato peels and pomegranate peel powder using 70% ethanol and characterized for their total phenolic, total flavonoid content, antioxidant capacity, and antimicrobial activity. Pomegranate peel extract exhibited the highest total phenolic content (382.07 mg equivalent gallic acid/g) and the total flavonoid content (144.85 mg equivalent quercetin/g), demonstrating superior antioxidant and antimicrobial activities compared to potato peel extract. The incorporation of potato peels and pomegranate peel powder into chicken meatballs significantly improved cooking yield while reducing the values of lipid oxidation (TBA) and total volatile basic nitrogen (TVB-N), thus improving oxidative stability and microbiological quality throughout refrigerated storage compared to control. Although the pomegranate peel exhibited stronger antioxidant activity, the potato peels achieved superior sensory acceptability. In particular, while the 4% potato peel + 4% pomegranate peel combination achieved the greatest reduction in lipid oxidation, protein degradation and microbial growth, the combined treatment containing 2% pomegranate peel + 2% potato peel achieved comparable protective performance while better preserving sensory acceptability, indicating an additive-to-possibly-combined effect that outperformed the corresponding individual treatments at the same inclusion level in maintaining the overall physicochemical, microbiological and sensory quality of chicken meatballs during 20 days of storage at 4 ± 1 °C. These findings suggest that the combined use of potato peels and pomegranate peel powder is a promising natural preservation strategy to improve the quality of the product and extend the refrigerated quality of chicken meatballs. Further work should include challenge testing, controlled interaction analysis, and broader consumer evaluation.

## 1. Introduction

Chicken meat plays a vital role in human nutrition due to its high protein content and relatively low fat levels, making it a healthy dietary option [1]. It is affordable, especially for low-income groups, and is considered safer and healthier compared to many other meats. Consistently high in quality, low in saturated fats, and capable of being fortified with essential nutrients, chicken is a widely preferred food across the world [2].

Like other types of meat, fresh chicken is highly perishable with a short shelf life, even under refrigeration. Its quality and freshness decline mainly due to the growth of psychrotrophic microbials and physicochemical changes [3]. Because it contains proteins, amino acids, fats, vitamins, minerals and high moisture, chicken meat provides a favorable environment for microorganisms to survive and multiply during processing, storage, distribution and at the consumer stage [4].

Chicken meat is spoiled during storage due to microbial activity and enzymes, leading to breakdown of proteins and nitrogen compounds that produce toxic volatile amines (TVB-N), altering color, flavor, and acceptability. TVB-N levels increase with storage time, aligning with microbial growth. in addition to meat lipids becoming rancid because of oxidation, and oxidative rancidity occurs by microorganisms [5]. In this sense, traditional preservation methods extend self-life while maintaining stability and sensory quality, but growing consumer concerns over chemical additives, stricter European regulations, and the demand for sustainability have driven interest in natural alternatives. Natural additives such as antimicrobial and antioxidant agents could provide a sustainable solution to this problem [6,7,8]. Plants (herbs and spices), mushrooms, animals, enzymes, and microorganisms are considered reservoirs of the natural active components [9,10]. Agroindustrial by-products, such as fruit peels, vegetable residues, and seeds, are rich in flavonoids, polyphenols, and antioxidants with strong antimicrobial and antioxidant effects [11]. Bioactive compounds such as terpenoids, polyphenols, and alkaloids provide health benefits as a result of their anti-inflammatory and antioxidant properties [12,13,14]. They are present in many plant-based foods and beverages, including olives, tea, oil, chocolate, and fruit juices. Food waste from agriculture and the supply chain is also a valuable source of these bioactive compounds [15].

One of the main problems with environmental effects is food waste, which not only causes food loss, but also wastes resources such as water, energy, and fertilizer that were needed in its creation. Parts such as peels, roots and seeds, often discarded before retail, are rich in valuable compounds, including phytochemicals, vitamins, and minerals with health-promoting effects [16]. Transforming these wastes into therapeutic and functional sources offers a sustainable and low-cost alternative to disposal, allowing their recycling and reuse in the food industry [17]. In this regard, we find that there are numerous studies proving that many by-products of vegetables and fruits contain bioactive components such as onion skin, marjoram, potato peel, fennel, black seed, cinnamon, olive leaf, Grape Pomace, Apple Pomace, Citrus fruits peel, tomato pomace, banana peel, mango, beet pulp, olive endocarp, pineapple pomace, Sugarcane Bagasse, Pear leaves and pomace, Pea pod/hull—Incineration, Carrot pomace and peel [17,18,19,20].

Potato peels, often discarded, are rich in phenols, flavonoids, vitamin C, and anthocyanins, giving them strong antioxidant, antimicrobial, and anti-inflammatory properties that sometimes exceed the nutritional content of potato flesh [21]. Similarly, pomegranate fruits and their peels are abundant in phytochemicals such as phenols, flavonoids, and hydrolyzable tannins, which provide antioxidant, antimicrobial, anticancer, and anti-inflammatory benefits [22,23]. Pomegranate peel extracts (PPE) show particular promise in preventing food spoilage and improving storage capacity [24]. The bioactive content of potato and pomegranate by-products depends on variety, cultivation, and processing methods, making them valuable sources for health-promoting and food-preserving applications [25].

Pomegranate and potato peels can prevent lipid oxidation, limit microbial growth, and preserve sensory attributes [26]. The use of these by-products not only enhances food quality and self-life but also reduces environmental impact by valorizing waste. wherein the use of these ingredients in chicken meat products may allow the meat to age more slowly and maintained quality during refrigerated storage. Therefore, the objective of the study was to compare the use of potato peel powder and pomegranate peel powder to preserve refrigerated chicken meatballs, in addition to the benefits of combining the two powders together.

## 2. Materials and Methods

### 2.1. Materials

#### 2.1.1. Raw Materials

Potatoes (*Solanum tuberosum*), fresh pomegranates (*Punica granatum* L.), chicken breast meat, spices, salt, onion and wheat flour were purchased from the local market of Fayoum, Egypt.

#### 2.1.2. Media

LB broth and agar, total count agar, MacConkey agar and potato dextrose agar were prepared using the procedures outlined in [27]. All other chemicals were of analytical quality.

#### 2.1.3. Microorganisms

Representative indicator microorganisms were used to assess antimicrobial activity, including the Gram-positive bacteria *Bacillus subtilis* (ATCC6051) and *Staphylococcus aureus* (ATCC 13565), the Gram-negative bacterium *Escherichia coli* O157 (ATCC 1659) and the filamentous fungus *Aspergillus niger* (AUMC 3663). All test microorganisms were supplied as actively growing cultures by the Cairo Microbiological Resources Center (MIRCEN), Faculty of Agriculture, Ain Shams University, Cairo, Egypt.

### 2.2. Methods

#### 2.2.1. Preparation of the Peels Powders

Fresh potato was cleaned and peeled by hand. To remove the peels and seeds, the pomegranate fruits were carefully cut. After being cut into small pieces, the peels were dried for 12 h at 50 ± 5 °C in a hot air oven. After being cooled, the peels were ground in a laboratory grinder to a fine powder. After passing through a 60 mesh screen sieve, the powdered material was stored at room temperature (25 ± 5°C) in high-density polyethylene bags until needed.

#### 2.2.2. Chemical Composition of Peels

Ash, ether extract, moisture content, and crude protein were determined according to the methods of [28].

#### 2.2.3. Preparation of the Peel Extracts

Each peel powder (10 g) was extracted with 70% (*v*/*v*) ethanol by maceration for 24 h at room temperature. Extraction was performed at the ambient light with solid to solvent ration 1:10. The resulting mixtures were centrifuged at 10,000 rpm for 5 min and the supernatants were collected. The extraction process was repeated three times under identical conditions using the remaining residues to maximize extract recovery. The combined filtrates were concentrated using the rotary evaporator at 50 °C. The concentrated extracts obtained were stored at −20 °C until further analysis. The extraction yield (%) was determined based on the dry weight of the recovered extract relative to the initial weight of the peel powder.

#### 2.2.4. Determination of the Extraction Yield

The extraction yield of the peel extracts was calculated using the equation shown below.

$$\mathrm{Extraction}\;\mathrm{Yield}\;(\%)=\frac{W1}{W2}\times 100$$

where W1 is the weight of the extract after evaporation of the solvent and W2 is the dry weight of the peels.

#### 2.2.5. Determination of the Total Phenolic Content

Total phenols in the extracts prepared above were determined using the Folin-Ciocalteau colorimetric technique according to [29]. The assay used 0.2 mL of extract and 1 mL of Folin-Ciocalteu reagent. After 5 min, the reaction was stopped with 0.8 mL of 20% sodium carbonate and kept at room temperature in a dark place for 30 min. The absorbance was measured at 765 nm using an Ultraspect 2000 (Pharmacia Biotech, Uppsala, Sweden). The phenolic concentration was calculated using a standard curve established with a standard gallic acid solution, and the results were represented as mg gallic acid/g of sample (mg GAE/g).

#### 2.2.6. Determination of Flavonoid Contents

The spectrophotometric approach described by [30]. We were used to assessing the flavonoid content of extracts. To 1 mL of the sample extract sub-solution, 1 mL of 2% AlCl_3_ ethanol solution was added. The absorbance at 420 nm was measured after one hour at room temperature. Flavonoids were denoted by a yellow color. The total flavonoid content was calculated as quercetin (mg/g dry weight).

#### 2.2.7. Determination of DPPH Radical Scavenging Activity

The peel extract’s DPPH free radical scavenging activity was measured using the method described by [31]. The radical stock solution, generated by dissolving 50 mg DPPH in 1000 mL of methanol, was stored in the refrigerator for future use. In a test tube, 100 µL extracts were combined with 3.9 mL of DPPH working solution then stored in the dark for 30 min, the absorbance was measured at 517 nm. Methanol was used as a blank. The percentage of inhibition was calculated as follows.

% inhibition = (Abs_control_ − Abs_sample_ × 100)/Abs_control_

The antioxidant activity of the extracts was expressed as IC_50_, corresponding to the concentration required to inhibit 50% of the initial DPPH radicals.

#### 2.2.8. Antimicrobial Activity of Peel Extracts

The antibacterial activity of the extracts against *Bacillus subtilis*, *Escherichia coli*, and *Staphylococcus aureus* was evaluated using the agar well diffusion method according to Perveen method [32]. Bacterial suspensions were adjusted to a concentration of 1 × 10^6^ CFU/mL and uniformly inoculated onto the appropriate agar medium. Wells of 7 mm − diameter were prepared in the agar plates. The extracts were prepared at a concentration of 200 mg/mL, and aliquots of 20 µL were added to the wells. For the negative control, 10% DMSO, which was used as the extraction solvent, was added to the wells instead of the extract. The plates were incubated at 35 °C for 24 h for *B. subtilis* and at 37 °C for 48 h for *S. aureus* and *E. coli*. The inhibition zones were measured in millimeters.

For antifungal activity. The antifungal activities of peel extracts were tested against *Aspergillus niger* ATCC (AUMC3663), via the agar well diffusion method. Fungal cultures were initially incubated in potato dextrose broth (PDB) at a concentration of 1 × 10^7^ spores/mL for 5 days at 26 °C. Subsequently, 20 μL of extracts were added to 7 mm wells in potato dextrose agar (PDA) media. The plates were incubated for another 5 days at 26 °C.

#### 2.2.9. Preparation of Chicken Meatball

Making meatballs refers to the method of [33]. The meatball components (chicken breast meat 75%, egg 8%, wheat flour 3%, salt 1.5%, spices 0.5%, onion 4%, and soybean oil 8%) were mixed well and then mixed with dried peels at 6 levels. The dough was rolled and formed into a ball. The chicken meatballs were stored in refrigerated conditions (3 °C ± 1 °C) for 20 days. The control formulation received no peel powder addition and was otherwise mixed, shaped, cooked and stored identically to the treated formulations.

Added rates of plant peel powder to chicken meatballs:

(T1) Potato peel powder 2%

(T2) Potato peel powder 4%

(T3) pomegranate peel powder 2%

(T4) pomegranate peel powder 4%

(T5) 2% potato peel + 2% pomegranate peel

(T6) 4% potato peel + 4% pomegranate peel

#### 2.2.10. Determination of the Chemical Composition of Chicken Meatballs

Moisture content and Ash content were determined according to the procedure provided by [34].

The total nitrogen content (TN) was determined using the Kjeldahl method as described in the [34]. Protein content was calculated using the following equation: Protein content (%) = total nitrogen × 6.25.

The ether extract was extracted with petroleum ether (40–60 °C) using the AOAC Soxhlet apparatus. (2005).

Total carbohydrate content was calculated by difference according to the following equation: Carbohydrates (%) = 100 − (% protein + % Moisture + % Fat + % ash)

#### 2.2.11. Determine the Cooking Yield

The cooking yield was determined by measuring the weight of the chicken meatballs before and after cooking. The samples were cooked in a preheated oven at 200 °C for 15 min, and the cooking yield was calculated using the following equation:

Cooking yield (%) = (Weight after cooking/Weight before cooking) × 100

#### 2.2.12. Antioxidant Activity

The antioxidant activity of the meatball samples was evaluated using the DPPH radical scavenging assay according to the method described by [35]. Briefly, 1 g of each chicken meatball sample was weighed and extracted with 5 mL of methanol in a test tube for 24 h. Subsequently, 0.4 mL of the methanolic extract was mixed with 3.6 mL of DPPH solution. The reaction mixture was incubated in the dark at room temperature for 30 min to allow the reaction to proceed. The absorbance was then measured at 517 nm using a spectrophotometer. The percentage of DPPH radical scavenging activity was calculated using the equation:

DPPH inhibition (%) = [(A_control_ − A_sample_)/A_control_] × 100

where A_control_ and A_sample_ represent the absorbance values of the control and sample, respectively. The IC_50_ value was subsequently estimated as the concentration of extract required to achieve 50% DPPH radical inhibition and was calculated from the dose–response curve generated by plotting inhibition percentage against extract concentration.

#### 2.2.13. Sensory Evaluation of Chicken Meatballs

Organoleptic properties, that is, color, flavor, mouth feel, juiciness, and overall acceptability of chicken meatball samples, were evaluated after cooking. The sensory evaluation of the samples was carried out according to the method described by [36] utilizing the subsequent numerical scale: Outstanding 8.5; excellent 7–8.4; good 6–6.9; fair 5–5.9; very poor 3.9; poor 4–4.9.

#### 2.2.14. Evaluation of the Quality of Chicken Meatballs in Cold Storage

Over the course of 20 days, various analyses were carried out to evaluate the quality of chicken meatballs, including measurements of pH levels, reactive compounds of thiobarbituric acid (TBARS), and total volatile basic nitrogen values (TVBN). For a complete evaluation, microbiological analyzes were also carried out. Chicken meatballs were kept in a refrigerator at 4 °C ± 1 °C while data was collected every five days.

#### 2.2.15. Chemical Quality Attribute

Lipid oxidation was evaluated by determining the value of reactive thiobarbituric acid (TBARS) value according to the method of [37]. In summary, a mechanical homogenizer was used to homogenize 10 g of each sample with 50 mL of distilled water for two minutes. After moving the homogenate to a distillation flask, 2.5 mL of 4 N HCl (pH 1.5) and 47.5 mL of distilled water were added. The mixture was distilled and 50 mL of distillate was collected. Subsequently, 5 mL of the distillate was mixed with 5 mL of TBA reagent (0.2884 g TBA dissolved in 100 mL of 90% glacial acetic acid) in a stopper glass tube. The reaction mixture was heated in a boiling water bath for 35 min and then cooled to room temperature. The absorbance was measured at 538 nm using a UV-vis spectrophotometer. A blank was prepared using 5 mL of distilled water instead of sample distillate. The TBARS value was expressed as mg malondialdehyde (MDA)/kg of sample using the following equation:

TBARS value (mg MDA/kg sample) = Absorbance_538_ × 7.8

The total volatile basic nitrogen (TVB-N) content was determined using the magnesium oxide distillation method (Okoloff’s method) according to the [34]. The TVB-N levels per 100 g of material were used to express the results.

The pH of the chicken meatball samples was tested according to [38]. Briefly, 5 g of each sample was homogenized with 50 mL of distilled water, and the pH was determined at room temperature using a calibrated digital pH meter.

#### 2.2.16. Microbiological Examination

Microbiological quality was assessed by enumerating total viable bacteria, coliforms, and yeasts and molds using standard plate count procedures. For each analysis, 10 g of chicken meatball sample was aseptically transferred into 90 mL of sterile peptone water and homogenized thoroughly. Serial tenfold dilutions were then prepared, and 1 mL aliquots from the appropriate dilutions were plated in duplicate onto the corresponding culture media. Total viable bacterial were enumerated on Plate Count Agar (PCA) following incubation at 37 °C for 48 h, while coliforms were enumerated separately on MacConkey following incubation at 37 °C for 24 h, consistent with the media prepared in Section 2.1.2. Yeast and mold populations were determined by spreading 1 mL of the selected dilutions onto Potato Dextrose Agar (PDA) and incubating the plates at 25 °C for 3–5 days. Microbial colonies were counted after the incubation period, and the results were reported as logarithmic colony-forming units per gram of sample (log CFU/g).

#### 2.2.17. Statistical Analysis

The experiment was carried out using a randomized complete block design (RCBD), with treatments considered as fixed effects and the ten panelists serving as blocks [39]. Data were subjected to analysis of variance (ANOVA) using the general linear model (GLM) approach performed in InfoStat software (Version 2012). When significant differences were identified (*p* < 0.05), treatment means were separated using Duncan’s multiple range test at the level of significance of 5%.

## 3. Results and Discussion

### 3.1. Chemical Composition of Potato and Pomegranate Peel Powder (% Dry Weight)

Table 1 presents the findings of the chemical composition analysis, which evaluated moisture, ether extract, and ash. The potato peel was characterized by a moisture, ash and fat content of 7.80%, 6.31%, and 2.12%, respectively. Meanwhile, the pomegranate peel showed a higher moisture content (11.04%) but a lower ash content (3.01%) and a lower fat content (1.19%). The data obtained agreed with values previously documented in earlier studies by [40,41].

### 3.2. Extraction Yield

The percentage of extracts was calculated on the basis of the mass of dry plant material used. The percent of extract present is shown in Table 1. The percent rate of potato and pomegranate peel extract was 9.6% and 33.3%, respectively.

It is noted that the percentage of the extract from the pomegranate peels is higher than that from potatoes. peel extract, the higher extraction yield obtained from the pomegranate peel compared to the potato peel may be attributed to differences in its chemical composition and structural characteristics. Pomegranate peel is considered a rich source of extractable bioactive compounds, particularly phenolic compounds, hydrolyzable tannins, flavonoids and ellagic acid, which exhibit high solubility in polar solvents, resulting in increased extraction yield [42,43].

In contrast, potato peel contains relatively higher amounts of structural polysaccharides such as cellulose, hemicellulose, starch residues, and lignin, which are less soluble during conventional solvent extraction, thus reducing extract recovery [44]. Furthermore, the dense lignocellulosic matrix of potato peel can limit the penetration of the solvent and the mass transfer efficiency, leading to lower extraction yield compared to the pomegranate peel [45,46].

### 3.3. Total Phenols in Plant Peel Extracts

Total phenolic contents of the potato and pomegranate peel extract were quantified using Folin-Ciocalteu and AlCl_3_ assays, respectively. As detailed in Table 1, the pomegranate peel extract exhibited a higher phenolic content (382.07 mg GAE/g) compared to the potato peel extract (134.31 mg GAE/g).

Previous investigations have reported similar values for the total phenolic content (TPC) of pomegranate peel extracts [47], while [48] observed a wide variation in TPC values, ranging from 18 to 510 mg/g dry weight, depending on the characteristics of the sample and extraction conditions.

In this study, the total phenolic content (TPC) of the potato peel extract (134.31 mg GAE/g) exceeded the value reported by [49]. However, this value agreed with that reported by [50]. Such differences could be explained by variations in the drying process, as bioactive constituents are particularly sensitive to heat-induced degradation. Furthermore, TPC and antioxidant capacity are influenced by multiple intrinsic and extrinsic factors, including cultivar, geographical region, pretreatment conditions, processing temperature, storage environment, exposure to light and oxygen, as well as seasonal variations during sample collection [51].

Phenolics are widely recognized for their anti-inflammatory, antimicrobial, antiviral, and anticancer effects. Furthermore, they function as robust antioxidants by chelating metal ions, neutralizing free radicals, and enhancing the endogenous antioxidant defense system [52].

### 3.4. Total Flavonoids in Plant Peel Extracts

Similarly, flavonoid concentrations were higher in pomegranate peel extract (144.85 mg of quercetin/g) than in potato peel extract (16.06 mg of quercetin/g). The flavonoids content of the Pomegranate peel extract in our work was higher than that obtained by [53], who found that the total flavonoids of the Pomegranate peels were 48.75 mg QE/g. The flavonoid content obtained for potato peels in the current investigation exceeded the values documented by [54,55], while it was less than in the study by [56], indicating possible variations related to extraction conditions and sample characteristics. Flavonoids have a wide range of chemical and biological characteristics that offer substantial antimicrobial, antiviral, antioxidant, and antispasmodic activities [57,58].

### 3.5. DPPH Radical Scavenging Activity

The DPPH assay is commonly used to determine the free radical scavenging activity of antioxidant compounds. The principle of this assay is based on the capacity of the antioxidants present in plant extracts to donate hydrogen atoms, resulting in the reduction of DPPH radicals. This reduction is accompanied by a visible color change from purple to yellow, corresponding to the formation of the reduced form (DPPH-H). The IC50 value is defined as the concentration of the sample required to achieve a 50% inhibition of DPPH radicals and serves as an important parameter to assess antioxidant efficiency.

The antioxidant activity of the pomegranate and potato peel extracts was assessed using the DPPH radical scavenging activity and the findings are displayed in Table 1. The findings showed that at 1 mg/mL, pomegranate peels exhibited the lowest DPPH scavenging activity (inhibition of 91.84% with EC_50_ 0.40 mg/mL), followed by potato peels (49.40% with EC_50_ 1.25 mg/mL), it is evident from the results obtained that there is a positive relationship between the extracts of phenolic compounds and the effect on DPPH. A positive association between the inhibition capacity and total phenolic content (TPC) has been widely reported in extracts derived from different agricultural wastes [59]; the current results confirmed this trend.

### 3.6. Antimicrobial Activity of Potato and Pomegranate Peel Extracts

**Figure 1:**
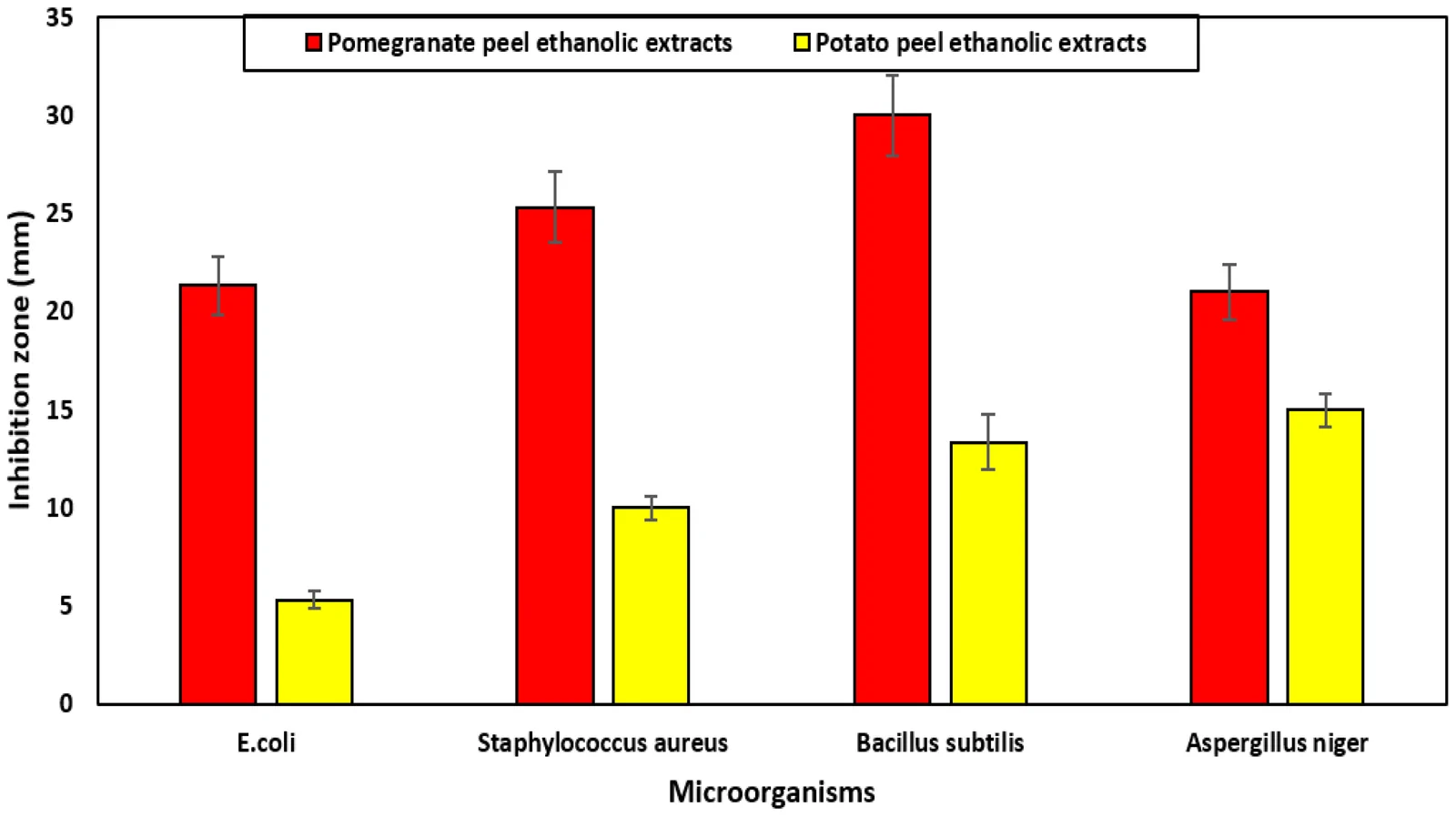
Antimicrobial Activity of Potato and Pomegranate Peel. Values are means ± SD of three replicates (*n* = 3).

The antimicrobial activity of different concentrations of potato peels and pomegranate peel extracts were measured using an agar well diffusion method and the results are presented in Figure 1. The results revealed that at 200 mg/mL, the potato peel results indicated that the lower inhibition zone in *E. coli* (5.33 mm) was more sensitive (13.33 mm) followed by *Staphylococcus aureus* (10.00 mm). The pomegranate peels also showed the highest inhibition zone in *B. subtilis* (30.00 mm), followed by *Staphylococcus aureus* (25.00 mm), then *E. coli* (21.33 mm). Both extracts have individually inhibited the fungal strain; *Aspergillus niger* (15.00 mm and 21.00 mm) for potato peels and pomegranate peel extracts, respectively.

These findings suggest that the pomegranate peel extract has a superior antimicrobial potential compared to the potato peel extract. Furthermore, both extracts demonstrated greater effectiveness against Gram-positive bacterial strains, while Gram-negative bacteria exhibited comparatively higher resistance. These findings are consistent with those reported by [54], who demonstrated that potato peel extract possesses antibacterial activity, with a more pronounced inhibitory effect against Gram-positive bacteria than against Gram-negative bacteria. Furthermore, Ref. [60] found that phenolic compounds extracted from potato peels and pulp significantly inhibited the growth of *Bacillus subtilis* infection. Furthermore, previous studies conducted by [61] reported that the ethanolic extract of potato peels exhibited significant antifungal activity against *Aspergillus niger*, further supporting the antimicrobial potential of the bioactive compounds of potato peel.

Furthermore, numerous studies reported that the ethanolic extract exhibits significant inhibitory activity against Gram positive and Gram-negative bacteria [62,63]. Furthermore, previous studies by [64] reported that the ethanolic extract of pomegranate peels exhibited significant antifungal activity against *Aspergillus niger*, supporting its effectiveness as a promising natural antimicrobial source.

### 3.7. Chicken Meatballs Fortified with Pomegranate Peel and Potato Peel Powder

#### 3.7.1. Chemical Composition of Chicken Meatballs

The approximate composition analysis was performed, including protein, moisture, ether extract, ash, and carbohydrate content, and the results obtained are presented in Table 2. The moisture content of all meatball formulations ranged from 61.11% to 64.13%. The ash content varied between 2.52% and 3.41% between the different treatments. At the initial stage, the protein content ranged from 16.75% to 18.31%, while the ether extract varied from 11.22% to 12.03%. Additionally, the carbohydrate content of the meatball samples ranged between 3.58% and 6.76%.

Incorporation of pomegranate and potato peel powder resulted in a slight increase in ash content, which could be attributed to the relatively high mineral content naturally present in both peel powders, as previously shown in Table 1. A notable increase in the carbohydrate percentage was also observed in the treated samples containing both peel powders. This increase may be explained by the relatively higher carbohydrate content of pomegranate peel and potato peel powders, as reported similarly by [21,42].

#### 3.7.2. Cooking Yield

Cooking yield is considered an important quality parameter in meat products because it reflects product behavior during thermal processing, particularly the interactions between meat components and incorporated non-meat ingredients, such as macromolecular hydrocolloids, fiber, and starches which are known to have water binding capacity, on the treated products. As presented in Table 2, the percentage of cooking yield was evaluated to determine the effect of the pomegranate peel powder and the potato peel powder on both control and treated meatball samples formulated with different concentrations of peel powders.

The results demonstrated a gradual increase in cooking yield with increasing levels of both pomegranate peel powder and potato peel powder incorporated into the meatball formulations. In comparison, samples containing potato peel powder exhibited lower cooking loss than samples containing only pomegranate peel powder. Furthermore, treatments formulated with a combination of both peel powders showed improved cooking performance, particularly the 8% treatment, which recorded the highest cooking yield value (89.54%).

The improvement in cooking yield observed in the treated meatball samples can be attributed to the dietary fiber present in the pomegranate peel and the potato peel powders. Dietary fiber has a high-water holding capacity, which enhances moisture retention during thermal processing and, consequently, reduces cooking losses, leading to an improved cooking yield [65]. These findings are consistent with those reported by [66], who similarly reported that adding pomegranate peels to chicken meatballs improved their ability to retain water, thus increasing cooking yield. They are also consistent with the findings of [67], which reported that adding potato peel to chicken burgers improved water retention and increased cooking time.

#### 3.7.3. Antioxidant Scavenging Activity of Chicken Meatball Samples

The DPPH radical scavenging assay, a commonly used technique to assess the free radical scavenging ability of antioxidant compounds, was used to assess the antioxidant activity of chicken meatball samples. The DPPH radical, a persistent nitrogen-centered free radical with a deep purple hue, is frequently used to evaluate the antioxidant capacity of food samples and plant extracts [68].

The DPPH radical scavenging activity of meatball formulations during the initial storage period is presented in Table 2. The results demonstrated that all treated samples exhibited significantly higher antioxidant activity than the control sample. Among the treatments, containing 4% potato peel + 4% pomegranate peel, the highest free radical scavenging capacity (92.11%), while the control treatment exhibited the lowest antioxidant activity (26.58%).

Furthermore, meatball formulations supplemented with pomegranate peel powder showed greater antioxidant activity than those containing only potato peel powder. Interestingly, the combined incorporation of both peel powders resulted in the highest antioxidant activity among all treatments, indicating a possible combined interaction between their bioactive compounds, which increased the overall antioxidant capacity of the formulations.

The superior antioxidant activity observed in the treatments, which contain 4% potato peel, with 4% pomegranate peel can be attributed to the high concentration of phenolic compounds and flavonoids present in pomegranate and potato peel, which are well known for their hydrogen donating capacity and effective free radical scavenging properties. Furthermore, the results suggest that increasing the level of natural antioxidant sources improves the oxidative stability of meat products [24].

#### 3.7.4. Sensory Evaluation

**Figure 2:**
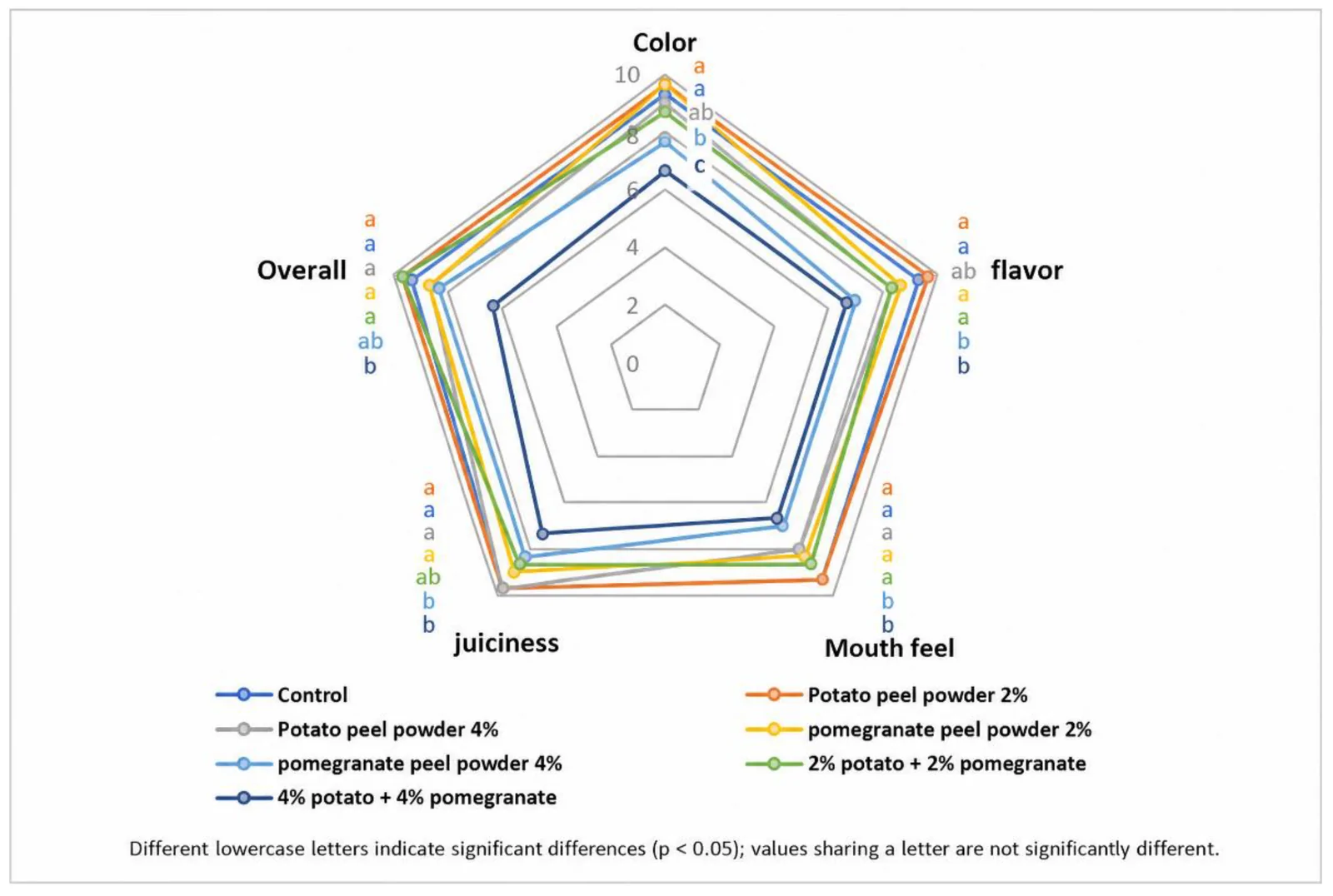
Sensory characteristics of chicken meatball samples. Different lowercase letters indicate significant differences (*p* < 0.05); values sharing a letter are not significantly different.

The sensory properties of the chicken meatball formulations were evaluated, and the obtained data were subjected to statistical analysis. The results are presented in Figure 2, where higher sensory scores indicate greater product acceptability.

Regarding color, no significant differences (*p* > 0.05) were observed between the control sample and formulations containing 2% potato peel powder or 2% pomegranate peel powder. However, increasing the level of incorporation to 4%, individually or in combination, resulted in a significant reduction (*p* < 0.05) in color acceptability. For flavor, no significant differences (*p* > 0.05) were detected between the control, 2% potato peel powder, 2% pomegranate peel powder, and the combined treatment containing 4% peel powder. On the contrary, the formulations containing 4% pomegranate peel powder and the combined treatment containing 8% peel powder received significantly lower flavor scores (*p* < 0.05).

In terms of mouthfeel, no significant differences (*p* > 0.05) were observed between most treatments. However, the samples supplemented with 4% pomegranate peel powder and the combined treatment of 8% exhibited relatively lower mouthfeel scores than the other formulations. Regarding juiciness, all treatments showed sensory scores comparable to those of the control sample, except for the combined 8% treatment, which recorded a significantly lower juiciness score (*p* < 0.05).

Regarding overall acceptability, no significant differences (*p* > 0.05) were observed between the control sample and the formulations containing 2% or 4% potato peel powder, as well as the formulation containing 2% pomegranate peel powder. Likewise, the meatball formulation containing the 4% combined mixture of potato and pomegranate peel powders exhibited general acceptability.

#### 3.7.5. Thiobarbituric Acid of Chicken Meatball Samples

The data in Table 3 showed the effect of the pomegranate peel powder and potato peel powder on the TBA value in chicken meatballs during refrigerated storage (4 ± 1 °C) for 20 days. There was a gradual increase in TBA values for all samples examined during the storage period. Non-differences were observed between treated chicken meatballs and control samples at zero time. After 20 days of refrigerated storage, the control sample exhibited the highest TBA value (0.89 mg MD/kg), indicating extensive lipid oxidation. TBA values, reaching 0.36, 0.32, 0.32, 0.22, 0.22, and 0.18 mg MD/kg for formulations containing 2% potato peel powder, 4% potato peel powder, 2% pomegranate peel powder, 4% pomegranate peel powder, 2% potato peel + 2% pomegranate peel powder and 4% potato peel + 4% pomegranate peel powder, respectively.

In general, storage time had an influence on the development of lipid oxidation in all the samples investigated. However, the development rates of the TBA values in the pomegranate peel powder and potato peel powder were very slow. Furthermore, the combined incorporation of both peel powders provided the greatest protection against protein deterioration, with the 4% potato peel + 4% pomegranate peel treatment maintaining the lowest TVBN value throughout storage. This enhanced preservative effect is likely to be attributed to the combined action of the phenolic compounds and other bioactive constituents present in both peel powders, which possess strong antioxidant properties capable of suppressing lipid degradation. Phenolic compounds are well recognized for their antioxidant properties, as they inhibit the formation and propagation of free radicals through their ability to chelate transition metal ions, particularly iron, thus reducing oxidative reactions [11]. This antioxidant mechanism contributes to improved oxidative stability and delayed quality deterioration in meat products. The present findings are consistent with those reported by [66,67].

#### 3.7.6. Total Volatile Bases Nitrogen of Chicken Meatball Samples

The effect of pomegranate peel powder and potato peel powder on the total volatile basic nitrogen (TVBN) content of chicken meatballs during refrigerated storage (4 ± 1 °C) for 20 days is presented in Table 3. TVBN is one of the most widely accepted indicators of meat freshness and protein deterioration, as it reflects the accumulation of volatile nitrogenous compounds, including ammonia and amines, produced through the enzymatic and microbial degradation of proteins [5].

As shown in Table 3, the TVBN values progressively increased throughout the storage period in the treatments. On day 0, TVBN values ranged from 8.90 to 9.80 mg/100 g among the different formulations. After 20 days of refrigerated storage, the control sample exhibited the highest TVBN value (25.70 mg/100 g), indicating extensive protein degradation. On the contrary, the treated samples showed significantly lower TVBN values, reaching 21.50, 20.20, 16.60, 11.00, 10.50, and 9.80 mg/100 g for the formulations containing 2% potato peel powder, 4% potato peel powder, 2% pomegranate peel powder, 4% pomegranate peel powder, 2% potato peel + 2% pomegranate peel powder and 4% potato peel + 4% pomegranate peel powder, respectively.

These findings clearly demonstrate that the incorporation of pomegranate peel powder and potato peel powder effectively retarded the formation of TVBN during refrigerated storage compared to the control treatment; this result agree with [67,69]. Furthermore, formulations containing pomegranate peel powder exhibited greater stability than those containing potato peel powder alone. The combined incorporation of both peel powders provided the greatest protection against protein degradation, with the 4% potato peel + 4% pomegranate peel treatment maintaining the lowest TVBN value throughout storage. This enhanced preservative effect is likely to be attributed to the combined action of phenolic compounds and other bioactive constituents present in both peel powders, which possess strong antioxidant and antimicrobial properties capable of suppressing microbial growth and delaying protein degradation.

The progressive increase in TVBN observed during storage is mainly associated with microbial deamination of amino acids and microbial reduction of trimethylamine oxide (TMAO) to trimethylamine (TMA), resulting in the accumulation of volatile basic nitrogen compounds [5,70].

#### 3.7.7. Changes in the pH Values of Chicken Meatballs During Storage

Data in Table 3 showed that meatballs containing 2% potato peel powder, 4% potato peel powder, 2% pomegranate peel powder, 4% pomegranate peel powder, 2% potato peel + 2% pomegranate peel powder, 4% potato peel powder + 4% pomegranate peel powder and control sample have a pH of 5.86, 5.79, 5.58, 5.37, 5.45, 5.20 and 5.88 at zero days and increase to a pH of 5.20, 5.28, 4.99, 5.18, 4.97, 5.14 and 4.95, respectively, on day 20. The results showed a slight decrease in pH values throughout the refrigerated storage period in all treatments. However, the rate of pH reduction was lower in the samples supplemented with pomegranate and potato peel powders than in the control, indicating their ability to retard quality deterioration during storage. The decrease in pH can be attributed to microbial metabolism and the fermentation of residual carbohydrates, leading to the production of organic acids. In addition, lipid hydrolysis and other biochemical changes that occur during storage can also contribute to the observed reduction in pH. As storage progressed, growth further influenced the physicochemical properties of the meatball samples. The lower pH decline in the treated samples may be associated with the antimicrobial activity of phenolic compounds present in the pomegranate and potato peel powders, which inhibited microbial proliferation and slowed spoilage. These findings are consistent with those reported by [19], who observed similar changes in pH during refrigerated storage of chicken meatballs supplemented with antioxidants based on oats.

#### 3.7.8. Microbiological Assessment

The effect of pomegranate peel powder and potato peel powder on the microbiology content of chicken meatballs during refrigerated storage (4 ± 1 °C) for 20 days is presented in Table 4.

The total count of bacteria in meatballs containing 2% potato peel powder, 4% potato peel powder, 2% pomegranate peel powder, 4% pomegranate peel powder, 2% potato peel + 2% pomegranate peel powder, and 4% potato peel + 4% pomegranate peel powder and control sample has a total count of bacteria of 2.23, 2.25, 2.25, 2.50, 2.36, 2.47 and 2.47 log cfu/g at zero day and increase to Total count bacteria of 4.38, 2.93, 3.61, 2.59, 2.62, 2.67 and 5.78 log cfu/g, respectively at the 20th day.

The total count of yeasts and molds in meatballs containing 2% potato peel powder, 4% potato peel powder, 2% pomegranate peel powder, 4% pomegranate peel powder, 2% potato peel + 2% pomegranate peel powder and 4% potato peel + 4% pomegranate peel powder and control sample has total yeast and molds count of 1.54, 1.49, 1.35, 1.24, 1.33, 1.31 and 1.38 log cfu/g at zero day and increase to the Total yeasts and mold count of 3.45, 2.34, 3.01, 1.89, 2.14, 1.58 and 3.97 log cfu/g, respectively, at the 20th day.

The total count of the coliform group in meatballs’ containing 2% potato peel powder, 4% potato peel powder, 2% pomegranate peel powder, 4% pomegranate peel powder, 2% potato peel + 2% pomegranate peel powder and control sample has Total coliform group count of 1.18, 1.11, 1.21, 1.24, 1.18, 1.20 and 1.58 log cfu/g at zero-day and increase to the Total coliform group count of 2.39, 1.84, 2.03, 1.39, 1.68, 1.31 and 3.26 log cfu/g, respectively at the 20th day.

The low initial values of total viable count, total yeast and mold, and total coliform group observed in both control and treated samples indicate that chicken meatballs were prepared under good hygienic conditions and appropriate handling practices during processing.

As presented in Table 4, the low initial values of total viable count, total yeast and mold, and total coliform group observed in both control and treated samples indicate that the chicken meatballs were prepared under good hygiene conditions and appropriate handling practices during processing. Microbial counts increased gradually during refrigerated storage in all treatments. However, the increase was significantly lower in samples containing 4% pomegranate peel powder, 2% potato peel + 2% pomegranate peel powder and 4% potato peel + 4% pomegranate peel powder compared to the control. This gradual increase in microbial counts during storage is expected and can be attributed to the proliferation of spoilage microorganisms, which contributes to quality deterioration through lipid oxidation and protein degradation.

The lower microbial counts observed in treated samples may be attributed to the antimicrobial properties of phenolic compounds and other bioactive constituents present in pomegranate and potato peels, as previously reported by several authors [26,64,66]. Furthermore, the antimicrobial activity demonstrated by the peel extracts against common foodborne pathogenic and spoilage microorganisms (Figure 1) supports their effectiveness in suppressing microbial growth throughout refrigerated storage. Consequently, the treated samples exhibited a slower rate of microbial increase over the 20-day storage period relative to the control, consistent with a possible extension of the microbial lag phase; however, formal microbial growth-kinetic modelling was not performed in the present study and is recommended in future work to confirm this interpretation.

The results clearly demonstrated that pomegranate peel powder exhibited greater antimicrobial activity than potato peel powder when applied individually, as evidenced by the lower total viable count, total yeast and mold count, and total coliform group count throughout the refrigerated storage period. This superior antimicrobial efficacy is probably attributable to the higher concentration of phenolic compounds and other bioactive constituents present in the pomegranate peel. Interestingly, the formulation containing a combination of 2% potato peel powder and 2% pomegranate peel powder showed greater antimicrobial effectiveness than the formulations containing 2% of either peel powder alone. This finding suggests a combined interaction between the bioactive compounds of both peel powders, resulting in enhanced inhibition of microbial growth. The complementary action of the phenolic compounds, flavonoids, and other natural antimicrobial constituents present in the pomegranate, and potato peels may have contributed to the improved preservative effect observed in the combined treatment.

## 4. Conclusions

The present study demonstrates that potato and pomegranate peel powders can serve as effective natural preservatives that improve the quality and support the preservation of chicken meatballs under refrigerated storage. Both peel powders improved cooking performance, reduced lipid oxidation and protein degradation, and slowed microbial growth during 20 days of storage at 4 ± 1 °C relative to the control. Pomegranate peel powder exhibited superior antioxidant and antimicrobial activity, attributable to its higher phenolic and flavonoid content, while potato peel powder provided greater sensory acceptability. In particular, the combined incorporation of 2% pomegranate peel powder and 2% potato peel powder achieved protective performance comparable to the higher-dose (4% + 4%) combination while better preserving sensory quality, suggesting an additive-to-possibly-combined effect that offered a favorable overall balance between preservation efficacy and consumer acceptability, though a dedicated statistical synergy/interaction analysis was not performed. These findings highlight the potential of combining potato and pomegranate processing by-products as a sustainable, clean-label preservation strategy that valorizes agro-industrial waste while supporting the quality and consumer acceptability of meat products. The composition of peel powders can vary with cultivar and processing conditions, and the study did not measure powder microbiological quality, pesticide residues, or potato glycoalkaloids; it also used a small sensory panel, did not evaluate relevant pathogens in the product, and did not include a formal synergy design. Accordingly, the findings should not be interpreted as proof of product safety, a quantified shelf-life extension. Future studies should use independently replicated production batches, validated matrix-specific microbiological methods, pathogen challenge testing where appropriate, standardized raw-material characterization, and a factorial interaction analysis.

## Figures and Tables

**Table 1:** Some chemical composition, extract, total phenols, total flavonoids, and antioxidant activity of potato and pomegranate peel powder (% dry weight).

Parameter	Potato Peel	Pomegranate Peel
**Moisture**	7.80 ± 0.45 ^b^	11.04 ± 1.02 ^a^
**Ash**	6.31 ± 0.80 ^a^	3.01 ± 0.02 ^b^
**Crude ether extract**	2.12 ± 1.20 ^a^	1.19 ± 1.12 ^b^
**Yield Extract%**	9.6 ± 0.09 ^b^	33.3 ± 0.65 ^a^
**Total Phenols** **(mg of gallic acid/g extract)**	134.31 ± 1.90 ^b^	382.07 ± 1.81 ^a^
**Total flavonoids** **(mg quercetin/g extract)**	16.06 ± 0.22 ^b^	144.85 ± 0.82 ^a^
**DPPH Radical scavenging activity (%)**	49.40 ± 0.69 ^b^	91.84 ± 0.88 ^a^
**IC_50_ (mg/mL)**	1.25 ± 0.96 ^a^	0.40 ± 0.50 ^b^

Values are means ± Standard deviation of three replicates. Mean values in the same row followed by different lowercase letters are significantly different according to Duncan’s multiple range test (*p* ≤ 0.05).

**Table 2:** Chemical composition, cooking loss, and antioxidant scavenging activity of chicken meatball samples.

Parameter	Control	Potato Peel Powder 2%	Potato Peel Powder 4%	Pomegranate Peel Powder 2%	Pomegranate Peel Powder 4%	2% Potato + 2% Pomegranate	4% Potato + 4% Pomegranate
**Moisture content**	64.13 ± 0.88 ^a^	63.84 ± 0.04 ^b^	62.92 ± 0.03 ^cd^	63.34 ± 0.24 ^bc^	62.32 ± 0.09 ^c^	62.30 ± 0.11 ^c^	61.11 ± 0.29 ^d^
**Ash**	2.52 ± 0.09 ^d^	2.58 ± 0.02 ^cd^	2.78 ± 0.02 ^c^	2.56 ± 0.01 ^cd^	2.75 ± 0.04 ^c^	3.19 ± 0.04 ^b^	3.41 ± 0.07 ^a^
**Crude protein**	18.31 ± 0.16 ^a^	17.74 ± 0.07 ^b^	17.16 ± 0.08 ^d^	17.53 ± 0.21 ^c^	17.32 ± 0.11 ^cd^	17.61 ± 0.23 ^bc^	16.75 ± 0.19 ^e^
**Crude ether extract**	11.46 ± 0.43 ^bc^	11.94 ± 0.08 ^b^	11.49 ± 0.37 ^bc^	12.03 ± 0.04 ^a^	11.87 ± 0.17 ^b^	11.22 ± 0.10 ^c^	11.97 ± 0.66 ^b^
**Carbohydrate**	3.58 ± 0.11 ^f^	3.9 ± 0.02 ^e^	4.65 ± 0.06 ^c^	4.54 ± 0.15 ^d^	5.74 ± 0.11 ^b^	5.68 ± 0.15 ^bc^	6.76 ± 0.07 ^a^
**Cooking yield**	79.16 ± 0.52 ^f^	87.06 ± 0.05 ^b^	88.89 ± 0.07 ^ab^	81.48 ± 0.39 ^e^	84.92 ± 0.15 ^d^	86.92 ± 0.15 ^c^	89.54 ± 0.40 ^a^
**% DPPH**	26.58 ± 0.72 ^g^	28.00 ± 0.32 ^f^	33.90 ± 0.14 ^e^	75.11 ± 0.10 ^d^	82.81 ± 0.20 ^c^	87.21 ± 0.10 ^b^	92.11 ± 0.27 ^a^

Total carbohydrates calculated by difference, values are means ± Standard deviation of three replicates. Mean values in the same row followed by different lowercase letters are significantly different according to Duncan’s multiple range test (*p* ≤ 0.05).

**Table 3:** Change in the TBA values, TVBN, and pH value of the chicken meatball samples during cold storage for twenty days.

Parameters	Storage Time (Days)	Treatments						
		Control	Potato Peel Powder 2%	Potato Peel Powder 4%	Pomegranate Peel Powder 2%	Pomegranate Peel Powder 4%	2% Potato + 2% Pomegranate	4% Potato + 4% Pomegranate
**TBA**	0	0.13 ± 0.01 ^q^	0.13 ± 0.02 ^q^	0.13 ± 0.02 ^q^	0.13 ± 0.01 ^q^	0.13 ± 0.02 ^q^	0.13 ± 0.01 ^q^	0.13 ± 0.01 ^q^
	5	0.28 ± 0.01 ^g^	0.13 ± 0.02 ^q^	0.13 ± 0.02 ^q^	0.22 ± 0.01 ^j^	0.14 ± 0.01 ^p^	0.16 ± 0.01 ^n^	0.13 ± 0.01 ^q^
	10	0.44 ± 0.02 ^c^	0.16 ± 0.01 ^n^	0.15 ± 0.01 ^o^	0.26 ± 0.02 ^h^	0.18 ± 0.01 ^m^	0.18 ± 0.01 ^m^	0.15 ± 0.01 ^o^
	15	0.57 ± 0.05 ^b^	0.25 ± 0.01 ^i^	0.30 ± 0.01 ^f^	0.26 ± 0.02 ^h^	0.21 ± 0.02 ^k^	0.20 ± 0.01 ^kl^	0.16 ± 0.01 ^n^
	20	0.89 ± 0.09 ^a^	0.36 ± 0.01 ^d^	0.32 ± 0.01 ^e^	0.32 ± 0.03 ^e^	0.22 ± 0.01 ^j^	0.22 ± 0.01 ^j^	0.18 ± 0.01 ^m^
**TVBN**	0	8.90 ± 0.70 ^o^	8.90 ± 0.70 ^o^	9.10 ± 0.70 ^n^	8.90 ± 0.50 ^o^	8.90 ± 1.40 ^o^	9.10 ± 0.70 ^n^	9.80 ± 0.00 ^m^
	5	15.20 ± 0.70 ^i^	11.00 ± 1.40 ^k^	10.50 ± 0.70 ^l^	9.10 ± 0.70 ^n^	9.60 ± 0.70 ^mn^	9.10 ± 0.70 ^n^	9.80 ± 0.00 ^m^
	10	20.10 ± 0.70 ^d^	17.30 ± 0.70 ^g^	12.00 ± 0.60 ^k^	9.15 ± 0.65 ^lm^	10.30 ± 0.00 ^lm^	9.10 ± 0.70 ^n^	9.80 ± 0.00 ^m^
	15	22.90 ± 0.70 ^b^	19.40 ± 1.40 ^e^	19.20 ± 0.40 ^f^	12.20 ± 0.40 ^j^	10.30 ± 0.00 ^lm^	9.10 ± 0.70 ^n^	9.80 ± 0.00 ^m^
	20	25.70 ± 0.70 ^a^	21.50 ± 0.70 ^c^	20.20 ± 0.80 ^ef^	16.60 ± 0.20 ^h^	11.00 ± 0.00 ^jk^	10.50 ± 0.70 ^l^	9.80 ± 0.00 ^m^
**pH**	0	5.88 ± 0.06 ^a^	5.86 ± 0.01 ^a^	5.79 ± 0.01 ^bc^	5.58 ± 0.02 ^d^	5.37 ± 0.03 ^g^	5.45 ± 0.05 ^f^	5.20 ± 0.02 ^ij^
	5	5.87 ± 0.11 ^a^	5.83 ± 0.01 ^b^	5.77 ± 0.06 ^c^	5.57 ± 0.01 ^d^	5.29 ± 0.04 ^h^	5.50 ± 0.04 ^ef^	5.21 ± 0.0 ^ij^
	10	5.39 ± 0.04 ^g^	5.56 ± 0.01 ^e^	5.75 ± 0.07 ^c^	5.49 ± 0.01 ^f^	5.29 ± 0.02 ^h^	5.46 ± 0.03 ^f^	5.18 ± 0.03 ^j^
	15	5.22 ± 0.09 ^i^	5.38 ± 0.02 ^g^	5.29 ± 0.06 ^h^	5.05 ± 0.04 ^m^	5.27 ± 0.03 ^hi^	5.12 ± 0.11 ^l^	5.16 ± 0.04 ^jk^
	20	4.95 ± 0.06 ^o^	5.20 ± 0.02 ^ij^	5.28 ± 0.06 ^h^	4.99 ± 0.05 ^mn^	5.18 ± 0.02 ^j^	4.97 ± 0.05 ^n^	5.14 ± 0.04 ^k^

Values are means ± Standard deviation of three replicates. Mean values in each parameter followed by a different lower-case letter are significantly different by Duncan’s least-significant difference test at *p* ≤ 0.05.

**Table 4:** Change in the microbial count (log_10_ cfu/g) of chicken meatball samples during cold storage for twenty days.

Parameters	Storage Time (Days)	Treatments						
		Control	Potato Peel Powder 2%	Potato Peel Powder 4%	Pomegranate Peel Powder 2%	Pomegranate Peel Powder 4%	2% Potato + 2% Pomegranate	4% Potato + 4% Pomegranate
Total count bacteria log^10^ cfu/g	0	2.47 ± 0.08 ^j–p^	2.23 ± 0.03 ^q^	2.25 ± 0.05 ^pq^	2.25 ± 0.08 ^pq^	2.50 ± 0.10 ^j–o^	2.36 ± 0.21 ^m–q^	2.47 ± 0.24 ^j–p^
	5	2.74 ± 0.26 ^g–j^	2.69 ± 0.17 ^h–j^	2.56 ± 0.15 ^i–n^	2.39 ± 0.09 ^l–q^	2.52 ± 0.32 ^i–o^	2.44 ± 0.21 ^k–q^	2.34 ± 0.17 ^n–q^
	10	3.54 ± 0.02 ^d^	3.04 ± 0.01 ^ef^	2.67 ± 0.10 ^h–k^	2.96 ± 0.09 ^ef^	2.31 ± 0.01 ^o–q^	2.42 ± 0.04 ^l–q^	2.39 ± 0.13 ^l–q^
	15	4.88 ± 0.02 ^b^	3.08 ± 0.03 ^e^	2.83 ± 0.17 ^f–h^	3.03 ± 0.03 ^ef^	2.36 ± 0.10 ^m–q^	2.55 ± 0.15 ^i–n^	2.58 ± 0.09 ^i–m^
	20	5.78 ± 0.09 ^a^	4.38 ± 0.01 ^c^	2.93 ± 0.05 ^e–g^	3.61 ± 0.06 ^d^	2.59 ± 0.11 ^i–m^	2.62 ± 0.08 ^h–l^	2.67 ± 0.14 ^h–j^
The total yeast and molds count log^10^ cfu/g	0	1.38 ± 0.10 ^p–t^	1.54 ± 0.06 ^o–s^	1.49 ± 0.21 ^o–s^	1.35 ± 0.04 ^q–t^	1.24 ± 0.02 ^t^	1.33 ± 0.03 ^r–t^	1.31 ± 0.01 ^st^
	5	1.88 ± 0.06 ^i–k^	1.66 ± 0.03 ^l–o^	1.79 ± 0.03 ^j–l^	1.59 ± 0.06 ^m–o^	1.58 ± 0.10 ^m–o^	1.50 ± 0.07 ^o–r^	1.32 ± 0.32 ^r–t^
	10	2.37 ± 0.24 ^e^	2.31 ± 0.16 ^ef^	1.86 ± 0.03 ^i–k^	2.10 ± 0.20 ^gh^	1.70 ± 0.03 ^k–n^	1.89 ± 0.01 ^ij^	1.50 ± 0.30 ^o–s^
	15	3.22 ± 0.10 ^c^	2.87 ± 0.03 ^d^	2.03 ± 0.02 ^hi^	2.27 ± 0.03 ^e–g^	1.74 ± 0.07 ^j–m^	1.98 ± 0.04 ^hi^	1.53 ± 0.18 ^n–q^
	20	3.97 ± 0.07 ^a^	3.45 ± 0.02 ^b^	2.34 ± 0.04 ^e^	3.01 ± 0.02 ^d^	1.89 ± 0.09 ^i–k^	2.14 ± 0.01 ^fh^	1.58 ± 0.02 ^m–o^
Total coliform group count log^10^ cfu/g	0	1.58 ± 0.05 ^f–i^	1.18 ± 0.10 ^l–n^	1.11 ± 0.01 ^n^	1.21 ± 0.03 ^l–n^	1.24 ± 0.04 ^k–n^	1.18 ± 0.03 ^l–n^	1.20 ± 0.04 ^l–n^
	5	1.79 ± 0.07 ^ef^	1.38 ± 0.01 ^i–l^	1.35 ± 0.15 ^j–m^	1.15 ± 0.03 ^mn^	1.28 ± 0.02 ^j–n^	1.32 ± 0.01 ^l–n^	1.23 ± 0.05 ^k–n^
	10	2.54 ± 0.18 ^bc^	1.44 ± 0.05 ^h–k^	1.31 ± 0.31 ^j–m^	1.22 ± 0.07 ^l–n^	1.20 ± 0.20 ^l–n^	1.49 ± 0.01 ^g–j^	1.24 ± 0.01 ^k–n^
	15	2.70 ± 0.08 ^b^	1.57 ± 0.06 ^g–i^	1.58 ± 0.05 ^f–i^	1.66 ± 0.13 ^e–g^	1.35 ± 0.04 ^j–n^	1.62 ± 0.09 ^f–h^	1.28 ± 0.02 ^j–n^
	20	3.26 ± 0.23 ^a^	2.39 ± 0.31 ^c^	1.84 ± 0.04 ^e^	2.03 ± 0.02 ^d^	1.39 ± 0.01 ^i–l^	1.68 ± 0.03 ^e–g^	1.31 ± 0.03 ^j–n^

Values are means ± Standard deviation of three replicates. Mean values in each parameter followed by a different lower-case letter are significantly different by Duncan’s least-significant difference test at *p* ≤ 0.05.

## Data Availability

The original contributions presented in the study are included in the article, further inquiries can be directed at the corresponding author.
